# Self-reported work-related accumulative fatigue of nurses: A cross-sectional study in public hospitals in China

**DOI:** 10.3389/fpubh.2022.1019092

**Published:** 2022-10-06

**Authors:** Changmin Tang, Xin Chen, Guangwen Gong, Cuiling Guan, Chaojie Liu

**Affiliations:** ^1^School of Management, Hubei University of Chinese Medicine, Wuhan, China; ^2^School of Psychology and Public Health, La Trobe University, Melbourne, VIC, Australia

**Keywords:** work-related accumulative fatigue, nurse, public hospital, cross-sectional survey, China

## Abstract

**Objectives:**

Work-related fatigue is a serious safety risk to nurses and their patients. This study aimed to assess self-reported work-related accumulative fatigue of nurses and its associated factors.

**Methods:**

A questionnaire survey of 2,918 clinical nurses conveniently sampled from 48 public hospitals across six provinces in China was conducted. The “*Self-diagnosis Checklist for Assessment of Workers' Accumulated Fatigue*” was adopted to assess the level of work-related accumulative fatigue of the study participants. Chi-square tests and ordinal regression analyses were performed to determine the sociodemographic characteristics associated with work-related accumulative fatigue.

**Results:**

About one third of respondents reported low work-related accumulative fatigue, compared with 23.1% reporting high and 24.6% reporting very high levels of work-related accumulative fatigue. Higher levels of work-related accumulative fatigue were associated with female gender (AOR = 0.614 for male relative to female, *p* = 0.005), age between 30 and 40 years (AOR = 1.346 relative to >40 years, *p* = 0.034), 5–10 years of work experience (AOR = 1.277 relative to >10 years, *p* = 0.034), and bachelor or above degree qualifications (AOR = 0.806 for associate degree relative to bachelor or above degree, *p* = 0.007). Those who worked in rural county hospitals (AOR = 0.816 for metropolitan relative to rural county hospitals, *p* = 0.006) and resided in central China (AOR = 1.276 relative to western China, *p* = 0.004) had higher odds of reporting higher levels of work-related accumulative fatigue.

**Conclusion:**

High levels of work-related accumulative fatigue are evident in nurses of public hospitals in China. The problem is more serious in the female nurses in their mid-career and those who worked in the central region and rural setting.

## Introduction

Fatigue of nurses is a safety risk for both nurses and patients (1, 2). It not only affects the health of nurses, but also increases the risk of medical errors (3–5). Fatigue is associated with anxiety, depression, poor quality of sleep (6), and increased sick leave (7). It jeopardizes the clinical performance of healthcare workers (8–10). Nurse fatigue was reported to have played a role in 83% of medical errors (11). Long night shifts are often blamed for inevitable fatigue of nurses (12–14). However, our understanding about work-related accumulative fatigue in nurses has been limited.

Nursing services are critical in all aspects of health care. As the largest health workforce, nurses have made great contributions to the maintenance of population health and ensuring global health security (15). However, the shortage of nursing staff has been a worldwide concern (16), which can impose excessive physical and mental loads on the practicing nurses (17). Many nurses have experienced accumulative fatigue and burnout, and eventually chosen to leave the healthcare industry, resulting in a vicious circle of workforce shortage (18–20).

Over the past few decades, China has experienced unprecedented rapid socioeconomic development, including in the health sector. However, like in many low- and middle-income countries (21), a sustained shortage of human resources in health has been a significant barrier for meeting the ever-increasing needs of consumers. The shortage of nursing workforce is particularly serious in China. Although the number of registered nurses more than doubled within 10 years, increasing from 1.85 million (1.39 per 1,000 population) in 2009 to 4.10 million (2.94 per 1,000 population) in 2018 (22), the nursing shortage problem persists in China (23). On average, the world has 4 nurses per 1,000 population in 2017, 15.7 in the United States, 12.7 in Japan, and 7.5 in Korea in 2018 (24). The doctor-nurse ratio in China reached 1:1.14 in 2018 (1: 1.15 in 2020), still lower than the target (1:2) set up by the Chinese government (22, 25).

There exist significant regional and setting disparities in the distribution of health workforce in China (26). In 2018, the number of registered nurses per 1,000 population reached 5.08 in urban facilities in China, compared with 1.80 in rural. The central region had the lowest number of nurses (2.7 per 1,000 urban and rural population) (22).

This study aimed to determine the prevalence of work-related accumulative fatigue in nurses in China's public hospitals and its associated factors.

## Methods

A cross-sectional survey of nurses in public hospitals was conducted. Ethics approval was granted by the Research Ethics Committee of Tongji Medical College, Huazhong University of Science and Technology (No: IORG0003571).

### Study setting

The study was conducted in the public hospitals sampled from both urban and rural settings across eastern, central, and western regions of China. In China, public hospitals account for more than 73% of hospital beds whilst employing more than 79% of registered nurses in 2018 (22). Consumers enjoy freedom to choose among different levels of medical institutions. Both urban and rural public hospitals are usually overcrowded (27, 28).

### Study sample

Study participants were recruited using a multi-stage stratified sampling strategy. Two provinces from the most developed eastern region (Shandong and Hebei), the developing central region (Hubei and Hunan), and the least developed western region (Guizhou and Qinghai) were purposely identified first, respectively. This was followed by convenient sampling of four metropolitan public tertiary hospitals and four rural county public hospitals in each participating province.

Clinical nurses who had direct patient contacts in the 48 sample hospitals were eligible for the survey. A sample size of 2,422 to 3,776 would enable detection of a difference of 2–2.5 percentage points should 25% respondents had a certain level of work-related accumulative fatigue (assuming an even distribution of respondents across the four levels of accumulative fatigue), with α being set at 0.05 and statistical power (1-β) being set at 0.80 (29). Therefore, we decided to recruit 80 nurses from each metropolitan tertiary hospital and 60 nurses from each rural county hospital.

### Survey instrument

The questionnaire was developed in Chinese language, which contained two sections. The first section tapped into the sociodemographic characteristics of respondents, including gender (male, female), age (years), marital status (married, not married), qualification (associate degree, bachelor degree or above), professional title (no title, junior, middle, senior), monthly salary (<5,000, 5,000–8,000, >8,000 Yuan), and years of work experience.

The second section measured work-related accumulative fatigue using the “*Self-diagnosis Checklist for Assessment of Workers' Accumulated Fatigue*” scale, which was developed by the Ministry of Health, Labor and Welfare of Japan, and had been adapted to the context of China and validated in a variety of settings (30–32). The scale contains 13 items measuring symptoms of fatigue (such as sleepy, irritation, tiredness, anxiety, and difficult to focus) and 7 items measuring work burden (such as overwork, irregular schedule, night shift, and break time). The fatigue symptoms were rated on a three-point frequency scale (0 = rarely; 1 = sometimes; 3 = often), resulting in a summed score that categories fatigue into four grades: I (0-4); II (5-10); III (11-20); IV (21-39) (32). The work burden items were rated on a two- or three-point scale, ranging from low (0) to high (1 or 3) burdens (Supplementary Table S1). A summed score was then calculated to categorize work burden into four levels: A (0); B (1-2); C (3-5); and D (6-15) (32). The subscales of fatigue symptoms and work burden had a Cronbach's alpha coefficient of 0.931 and 0.813, respectively, in this study, indicating good internal consistency.

The two subscales generated a matrix (Table 1) that classifies work-related accumulative fatigue into four levels: low, a little high, high, and very high (28).

**Table 1:** Classification of work-related accumulative fatigue.

**Fatigue symptoms**	**Level of work burden**			
	**A (0)**	**B (1,2)**	**C (3–5)**	**D (6–15)**
I (0–4)	low	low	a little high	high
II (5–10)	low	low	a little high	high
III (11–20)	low	a little high	high	very high
IV (21–39)	low	a little high	high	very high

### Data collection

Data were collected from January to November 2018. Trained investigators visited the participating hospitals and invited the eligible nurses to self-complete the questionnaire. The survey was anonymous. On average, each survey took 5 to 10 min to complete. Implied informed consent was obtained from each participant prior to the survey. Participation in the survey was completely anonymous and voluntary. A total of 3,246 questionnaires were returned, with 2,918 (86.85%) containing no missing values and being included in data analysis.

### Statistical analysis

Data were entered into EpiData 3.0 and analyzed using SPSS 19.0. A two-side *p*-value of < 0.05 was considered statistically significant.

The characteristics of study participants were described through frequency distributions and compared between urban and rural settings and across the three regions using Chi-square tests. We tested the urban-rural and regional differences in the frequency distributions of fatigue symptoms and work burden in the study participants first using Chi-square tests (Supplementary Table S2), before presenting the levels of work-related accumulative fatigue by the characteristics of study participants. Multivariate ordinal regression models were established to determine the sociodemographic characteristics associated with work-related accumulated fatigue.

## Results

### Characteristics of respondents

The vast majority of respondents were women (96.4%), younger than 40 years (89.0%), married at the time of the survey (74.3%), and obtained a university degree (68.2%). Most (58.4%) had a junior professional title, and earned <5,000 Yuan monthly salary (equivalent to 740 US dollar). About 44% had 5–10 years of work experience (Table 2).

**Table 2:** Characteristics of study participants (*n* = 2,918).

**Characteristics**	**Total *N* (%)**	**Region**, ***N*** **(%)**				**Hospital**, ***N*** **(%)**		
		**Eastern**	**Central**	**Western**	***p***	**Metropolitan**	**Rural country**	***p***
**Gender**								
Male	105 (3.6)	61 (6.4)	11 (1.2)	33 (3.3)	<0.001	47 (2.7)	58 (5.0)	0.001
Female	2,813 (96.4)	895 (93.6)	943 (98.8)	975 (96.7)		1,719 (97.3)	1,094 (95.0)	
**Age (years)**								
<30	1,344 (46.1)	403 (42.2)	478 (50.1)	463 (45.9)	0.009	800 (45.3)	544 (47.2)	0.127
30–40	1,251 (42.9)	434 (45.4)	387 (40.6)	430 (42.7)		754 (42.7)	497 (43.1)	
>40	323 (11.1)	119 (12.4)	89 (9.3)	115 (11.4)		212 (12.0)	111 (9.6)	
**Marital status**								
Married	2,169 (74.3)	711 (74.4)	700 (73.4)	758 (75.2)	0.652	1,268 (71.8)	901 (78.2)	<0.001
Not married	749 (25.7)	245 (25.6)	254 (26.6)	250 (24.8)		498 (28.2)	251 (21.8)	
**Qualification**								
Associate degree	929 (31.8)	296 (31.0)	276 (28.9)	357 (35.4)	0.007	447 (25.3)	482 (41.8)	<0.001
Bachelor degree or above	1,989 (68.2)	660 (69.0)	678 (71.1)	651 (64.6)		1,319 (74.7)	670 (58.2)	
**Professional title**								
No title	383 (13.1)	163 (17.1)	93 (9.7)	127 (12.6)	<0.001	237 (13.4)	146 (12.7)	0.005
Junior	1,703 (58.4)	507 (53.0)	570 (59.7)	626 (62.1)		991 (56.1)	712 (61.8)	
Middle	734 (25.2)	251 (26.3)	255 (26.7)	228 (22.6)		467 (26.4)	267 (23.2)	
Senior	98 (3.4)	35 (3.7)	36 (3.8)	27 (2.7)		71 (4.0)	27 (2.3)	
**Monthly salary (Yuan)**								
<5,000	1,774 (60.8)	600 (62.8)	677 (71.0)	497 (49.3)	<0.001	893 (50.6)	881 (76.5)	<0.001
5,000–8,000	1,040 (35.6)	322 (33.7)	260 (27.3)	458 (45.4)		777 (44.0)	263 (22.8)	
>8,000	104 (3.6)	34 (3.6)	17 (1.8)	53 (5.3)		96 (5.4)	8 (0.7)	
**Years of work experience**								
<5	802 (27.5)	279 (29.2)	239 (25.1)	284 (28.2)	0.192	500 (28.3)	302 (26.2)	<0.001
5–10	1,283 (44.0)	403 (42.2)	447 (46.9)	433 (43.0)		720 (40.8)	563 (48.9)	
>10	833 (28.5)	274 (28.7)	268 (28.1)	291 (28.9)		546 (30.9)	287 (24.9)	
Total	2,918 (100)	956 (32.8)	954 (32.7)	1,008 (34.5)		1,766 (60.5)	1,152 (39.5)	

The respondents from the more developed eastern region were older compared with their central and western counterparts. A higher percentage of male nurses from the eastern region and the rural setting participated in the survey. The rural participants were more likely to be married than their urban counterparts. The participants from the western region and the rural setting had lower levels of education and lower salary, although the rural participants had longer working experience (Table 2).

### Fatigue symptoms and work burden

About 19% of respondents reported grade IV fatigue symptoms. Those from the central and western regions (*p* < 0.001) were more likely to report higher levels of fatigue (Table 3).

**Table 3:** Fatigue symptoms and work burden reported by respondents (*n* = 2,918).

	**Total N (%)**	**Region**, ***N*** **(%)**			***p***	**Hospital**, ***N*** **(%)**		***p***
		**Eastern**	**Central**	**Western**		**Urban**	**Rural**	
**Fatigue symptoms**								
I (0–4)	428 (14.7)	161 (16.8)	121 (12.7)	146 (14.5)	<0.001	274 (15.5)	154 (13.4)	0.381
II (5-10)	774 (26.5)	288 (30.1)	226 (23.7)	260 (25.8)		466 (26.4)	308 (26.7)	
III (11–20)	1,160 (39.8)	354 (37.0)	411 (43.1)	395 (39.2)		700 (39.6)	460 (39.9)	
IV (21–39)	556 (19.1)	153 (16.0)	196 (20.5)	207 (20.5)		326 (18.5)	230 (20.0)	
**Work burden**								
A (0)	645 (22.1)	222 (23.2)	186 (19.5)	237 (23.5)	0.001	415 (23.5)	230 (20.0)	<0.001
B (1-2)	585 (20.0)	189 (19.8)	171 (17.9)	225 (22.3)		382 (21.6)	203 (17.6)	
C (3-5)	894 (30.6)	295 (30.9)	291 (30.5)	308 (30.6)		525 (29.7)	369 (32.0)	
D (6-15)	794 (27.2)	250 (26.2)	306 (32.1)	238 (23.6)		444 (25.1)	350 (30.4)	

Similarly, 27.2% of respondents reported level D work burden. Those from the central region (*p* = 0.001) and rural settings (*p* < 0.001) were more likely to report higher levels of work burden (Table 3). Higher levels of fatigue symptoms were associated with higher levels of work burden (Supplementary Table S3).

### Work-related accumulative fatigue

About one third of respondents reported low work-related accumulative fatigue, while 23.1% reported high and 24.6% reported very high levels of work-related accumulative fatigue (Table 4). Female gender (*p* < 0.001) and younger age (*p* = 0.002) were associated with higher work-related accumulative fatigue. Those who were married (*p* = 0.001), held a bachelor degree or above degree (*p* < 0.001), and had a professional title (*p* < 0.001) and over 5 years of work experience (*p* < 0.001) were more likely to report higher levels of work-related accumulative fatigue. Higher levels of work-related accumulative fatigue were also reported by the respondents from the central region (*p* < 0.001) and rural settings (*p* = 0.011).

**Table 4:** Sociodemographic characteristics associated with work-related accumulative fatigue (*n* = 2,918).

**Characteristics**	**Work-related accumulative fatigue**				***p***
	**Low**	**A little high**	**High**	**Very high**	
**Gender**					
Male	32 (30.5)	50 (47.6)	10 (9.5)	13 (12.4)	<0.001
Female	932 (33.1)	511 (18.2)	664 (23.6)	706 (25.1)	
**Age (years)**					
<30	483 (35.9)	242 (18.0)	299 (22.2)	320 (23.8)	0.002
30–40	358 (28.6)	257 (20.5)	304 (24.3)	332 (26.5)	
>40	123 (38.1)	62 (19.2)	71 (22.0)	67 (20.7)	
**Marital status**					
Married	673 (31.0)	432 (19.9)	515 (23.7)	549 (25.3)	0.001
Not married	291 (38.9)	129 (17.2)	159 (21.2)	170 (22.7)	
**Qualification**
Associate degree	361 (38.9)	159 (17.1)	215 (23.1)	194 (20.9)	<0.001
Bachelor degree or above	603 (30.3)	402 (20.2)	459 (23.1)	525 (26.4)	
**Professional title**
No title	190 (49.6)	54 (14.1)	78 (20.4)	61 (15.9)	<0.001
Junior	529 (31.1)	333 (19.6)	396 (23.3)	445 (26.1)	
Middle	219 (29.8)	151 (20.6)	168 (22.9)	196 (26.7)	
Senior	26 (26.5)	23 (23.5)	32 (32.7)	17 (17.3)	
**Monthly salary (Yuan)**					
<5,000	554 (31.2)	346 (19.5)	406 (22.9)	468 (26.4)	0.067
5,000–8,000	368 (35.4)	197 (18.9)	243 (23.4)	232 (22.3)	
>8,000	42 (40.4)	18 (17.3)	25 (24.0)	19 (18.3)	
**Years of work experience**
<5	330 (41.1)	141 (17.6)	162 (20.0)	169 (21.1)	<0.001
5–10	355 (27.7)	260 (20.3)	316 (24.6)	352 (27.4)	
>10	279 (33.5)	160 (19.2)	196 (23.5)	198 (23.8)	
**Hospitals**					
Metropolitan	616 (34.9)	345 (19.5)	402 (22.8)	403 (22.8)	0.011
Rural county	348 (30.2)	216 (18.8)	272 (23.6)	316 (27.4)	
**Region**					
Eastern	331 (34.6)	206 (21.5)	201 (21.0)	218 (22.8)	<0.001
Central	265 (27.8)	185 (19.4)	221 (23.2)	283 (29.7)	
Western	368 (36.5)	170 (16.9)	252 (25.0)	218 (21.6)	
Total	964 (33.0)	561 (19.2)	674 (23.1)	719 (24.6)	

The ordinal logistic modeling confirmed that male respondents (AOR = 0.614, *p* = 0.005) and those who had associate degree (AOR = 0.806, *p* = 0.007), held a junior title (AOR = 0.644, *p* = 0.046) or no title (AOR = 0.374, *p* < 0.001), and worked in metropolitan hospitals (AOR = 0.816, *p* = 0.006) had lower levels of work-related accumulative fatigue. By contrast, an age between 30 and 40 years (AOR = 1.346, *p* = 0.034), 5–10 years of work experience (AOR = 1.277, *p* = 0.034), and working in the central region (OR = 1.276, *p* = 0.004) were significant predictors of higher work-related accumulative fatigue (Table 5).

**Table 5:** Predictors of work-related accumulative fatigue-ordinal logistic regression modeling (*n* = 2,918).

**Variables**	**β**	**Standard deviation**	**Wald**	***p***	**Adjusted odds ratio (AOR)**	**95% confidence interval**	
						**Lower bound**	**Higher bound**
**Threshold**							
(Low)	−0.692	0.272	6.470	0.011			
(A little high)	0.133	0.271	0.241	0.624			
(High)	1.190	0.272	19.085	<0.001			
**Gender**							
Men	−0.488	0.174	7.823	0.005	0.614	0.436	0.864
Women	Reference						
**Age (years)**							
<30	0.282	0.174	2.648	0.104	1.326	0.944	1.864
30–40	0.297	0.141	4.479	0.034	1.346	1.022	1.774
>40	Reference						
**Marital status**							
Married	−0.038	0.098	0.147	0.702	0.963	0.795	1.167
Not married	Reference						
**Qualification**							
Associate degree	−0.216	0.079	7.404	0.007	0.806	0.690	0.941
Bachelor degree or above	Reference						
**Professional title**							
No title	−0.985	0.249	15.660	<0.001	0.374	0.229	0.608
Junior	−0.441	0.221	3.979	0.046	0.644	0.417	0.992
Middle	−0.220	0.200	1.208	0.272	0.802	0.542	1.188
Senior	Reference						
**Monthly salary (Yuan)**
<5,000	0.374	0.194	3.724	0.054	1.453	0.994	2.123
5,000–8,000	0.139	0.190	0.539	0.463	1.149	0.792	1.668
>8,000	Reference						
**Years of work experience**							
<5	−0.036	0.154	0.056	0.813	0.964	0.713	1.304
5–10	0.244	0.115	4.484	0.034	1.277	1.018	1.600
>10	Reference						
**Hospitals**							
Metropolitan	−0.203	0.073	7.676	0.006	0.816	0.707	0.942
Rural country	Reference						
**Region**							
Eastern	−0.001	0.084	<0.001	0.988	0.999	0.848	1.176
Central	0.244	0.084	8.347	0.004	1.276	1.082	1.506
Western	Reference						

## Discussion

Over 47% of study participants in our study reported high or very high levels of work-related accumulative fatigue. Although this level is comparatively lower than that reported by medical doctors in China (28), it is higher than those reported by employees in other sectors, such as enterprise workers and lawyers (30, 33). Nurses provide continuous care for patients. They usually have night shifts and need to keep high levels of alertness day and night, which often lead to sleep problems (34, 35). Previous studies found that night shifts are associated with increased fatigue (17, 36, 37). Mild fatigue may be imperceptible, but accumulative fatigue can have serious consequences, even death from overwork (38).

We found that female nurses experienced higher levels of work-related accumulative fatigue than male nurses. This result is consistent with the findings of previous studies (38). Worldwide, nursing workforce is dominated by women. The gender difference is a serious issue of concern (39). Women also take disproportional burden of care for family members, not only for children, but also for the elderly (40–42).

It appears that the nurses in their mid-career, in particular those aged between 30 and 40 years, had 5–10 years of work experience, and held a professional title experienced higher levels of work-related accumulative fatigue than others. The results are consistent with the findings of other studies conducted in various hospital units in China (43–45). This is likely to be a reflection of high levels of responsibilities assigned to the nurses in these categories. Unlike their more senior colleagues, mid-career nurses continue to take frontline clinical duties in addition to supervision and management roles. A previous study found that senior nurses with a professional title reported high levels of stress in emergency management (46).

There is evidence that nursing staff shortage is associated with higher levels of work-related accumulative fatigue. In our study, those from the central region and rural settings reported higher levels of work-related accumulative fatigue. Despite rapid socioeconomic development in China, regional disparities remain a great concern. Financing accountability has been delegated to local governments in China. Although the central government has prioritized the least developed western region for central financial subsidies, the central developing region has been struggling with limited financial capacities (47). Rural county hospitals play a pivotal role in the Chinese healthcare system (48). They not only lead the three-tier healthcare delivery system for rural residents, but also serve as a liaising platform for tertiary hospitals (all metropolitan) to reach rural consumers (49). From 2010 to 2018, patient visits to country hospitals increased by 73.16%, but the number of staff in county hospitals only increased by 71.43%. Nursing shortage is particularly serious in rural settings. There were 5.08 registered nurses per 1,000 urban residents, compared with 1.80 for rural residents in 2018 (22).

Multi-faceted strategies are needed to manage work-related accumulative fatigue of nurses (50). An early warning system can be established to monitor the level of work-related accumulative fatigue experienced by nurses. A fine balance between work productivity and staff well-being needs to be maintained. Indeed, work life balance has become one of the major tasks of human resources management (51, 52). Additional support to mid-career female nurses can be prioritized if the choice of staffing increase is not available. Meanwhile, increasing central policy attention should be paid to addressing the inequalities across regions and between urban and rural settings. Strengthening primary care can also help reduce hospital burdens (53).

This study has several limitations. The study adopted a cross-sectional design and no causal relationships should be assumed. Self-reported data were collected, which are subject to recall bias. Further studies are needed to determine the impacts of the high level of work-related accumulative fatigue on the well-being of nurses and the safety of patients.

## Conclusion

High levels of work-related accumulative fatigue are evident in public hospital nurses in China, in particular in those in their mid-career. Although this problem is widespread across regions and in both urban and rural settings, significant regional disparities exist. Nursing shortage is most serious in the central region and rural settings, and their nurses have suffered a higher level of work-related accumulative fatigue than others. These call for urgent policy attention as work-related accumulative fatigue is not only a matter of staff well-being, but also a matter of patient safety and quality of patient care.

## Data availability statement

The raw data supporting the conclusions of this article will be made available by the authors, without undue reservation.

## Ethics statement

The studies involving human participants were reviewed and approved by Ethics Committee of Tongji Medical College, Huazhong University of Science and Technology (No: IORG0003571). Written informed consent for participation was not required for this study in accordance with the national legislation and the institutional requirements.

## Author contributions

CT, CG, and CL designed the project. CT, XC, CG, and CL performed literature review and drafted the article. CT, XC, GG, and CG participated in data collection and data analyses. All authors have read and approved the final article.

## Funding

This research was funded by the National Natural Science Foundation of China (No. 71603077).

## Conflict of interest

The authors declare that the research was conducted in the absence of any commercial or financial relationships that could be construed as a potential conflict of interest.

## Publisher's note

All claims expressed in this article are solely those of the authors and do not necessarily represent those of their affiliated organizations, or those of the publisher, the editors and the reviewers. Any product that may be evaluated in this article, or claim that may be made by its manufacturer, is not guaranteed or endorsed by the publisher.
