# Supplementary material for: Self-reported work-related accumulative fatigue of nurses: A cross-sectional study in public hospitals in China
Source: Front Public Health. 2022 Oct 6;10:1019092. doi: 10.3389/fpubh.2022.1019092 (PMC9582430; doi:10.3389/fpubh.2022.1019092)
Supplement: Supplementary file 1 [file Table_1.DOCX]

**Table S1: Self-diagnosis Checklist for Assessment of Workers’ Accumulated Fatigue**

|  | Item | Score | | |
| --- | --- | --- | --- | --- |
|  |  | 0 | 1 | 3 |
| Fatigue symptom | | rarely | sometimes | often |
| 1 | Irritation |  |  |  |
| 2 | Anxiety |  |  |  |
| 3 | Restless |  |  |  |
| 4 | Depression |  |  |  |
| 5 | Cannot sleep |  |  |  |
| 6 | Feeling ill |  |  |  |
| 7 | Lack of concentration |  |  |  |
| 8 | Often make mistakes |  |  |  |
| 9 | Feeling very sleepy during work |  |  |  |
| 10 | Unmotivated |  |  |  |
| 11 | Exhaustion |  |  |  |
| 12 | Feeling tired when waking up in the morning |  |  |  |
| 13 | Get tired more easily than previously |  |  |  |
| Working condition in the last month | |  |  |  |
| 1 | Overwork over a one-month period | Little or appropriate | Much | Very much |
| 2 | Irregular work (e.g. sudden task) | Little | Much |  |
| 3 | Business travel | No or little | Much |  |
| 4 | Working at night | No or little | Much | Very much |
| 5 | Break time and resting facility | Satisfied | Dissatisfied |  |
| 6 | Mental burden of work | Little | Much | Very much |
| 7 | Physical burden of work | Little | Much | Very much |

**Table S2. Fatigue symptoms and work burden reported by respondents**

| **Characteristics** | **Fatigue symptoms** | | | | χ^2^ | P | **Work burden** | | | | χ^2^ | P |
| --- | --- | --- | --- | --- | --- | --- | --- | --- | --- | --- | --- | --- |
|  | Grade Ⅰ | Grade Ⅱ | Grade Ⅲ | Grade Ⅳ |  |  | A | B | C | D |  |  |
| Gender |  |  |  |  |  |  |  |  |  |  |  |  |
| Men | 16(15.2) | 55(52.4) | 25(23.8) | 9(8.6) | 40.722 | **<0.001** | 19(18.1) | 23(21.9) | 48(45.7) | 15(14.3) | 15.747 | **0.001** |
| Women | 412(14.6) | 719(25.6) | 1135(40.3) | 547(19.4) |  |  | 626(22.3) | 562(20.0) | 846(30.1) | 779(27.7) |  |  |
| Age (years) |  |  |  |  |  |  |  |  |  |  |  |  |
| <30 | 234(17.4) | 367(27.3) | 498(37.1) | 245(18.2) | 24.437 | **<0.001** | 310(23.1) | 286(21.3) | 390(29.0) | 358(26.6) | 24.775 | **<0.001** |
| 30-40 | 150(12.0) | 325(26.0) | 515(41.2) | 261(20.9) |  |  | 240(19.2) | 233(18.6) | 413(33.0) | 365(29.2) |  |  |
| >40 | 44(13.6) | 82(25.4) | 147(45.5) | 50(15.5) |  |  | 95(29.4) | 66(20.4) | 91(28.2) | 71(22.0) |  |  |
| Marital status |  |  |  |  |  |  |  |  |  |  |  |  |
| Married | 276(12.7) | 561(25.9) | 901(41.5) | 431(19.9) | 32.879 | **<0.001** | 452(20.8) | 433(20.0) | 687(31.7) | 597(27.5) | 9.416 | **0.024** |
| Not married | 152(20.3) | 213(28.4) | 259(34.6) | 125(16.7) |  |  | 193(25.8) | 152(20.3) | 207(27.6) | 197(26.3) |  |  |
| Qualification |  |  |  |  |  |  |  |  |  |  |  |  |
| Associate degree | 166(17.9) | 248(26.7) | 358(38.5) | 157(16.9) | 13.364 | **0.004** | 253(27.2) | 187(20.1) | 267(28.7) | 222(23.9) | 23.328 | **<0.001** |
| Bachelor degree or above | 262(13.2) | 526(26.4) | 802(40.3) | 399(20.1) |  |  | 392(19.7) | 398(20.0) | 627(31.5) | 572(28.8) |  |  |
| Professional title |  |  |  |  |  |  |  |  |  |  |  |  |
| No title | 103(26.9) | 112(29.2) | 117(30.5) | 51(13.3) | 73.020 | **<0.001** | 139(36.3) | 74(19.3) | 96(25.1) | 74(19.3) | 61.205 | **<0.001** |
| Early career | 219(12.9) | 466(27.4) | 673(39.5) | 345(20.3) |  |  | 344(20.2) | 334(19.6) | 535(31.4) | 490(28.8) |  |  |
| Mid-career | 96(13.1) | 174(23.7) | 319(43.5) | 145(19.8) |  |  | 143(19.5) | 156(21.3) | 224(30.5) | 211(28.7) |  |  |
| Senior | 10(10.2) | 22(22.4) | 51(52.0) | 15(15.3) |  |  | 19(19.4) | 21(21.4) | 39(39.8) | 19(19.4) |  |  |
| Monthly salary (yuan) |  |  |  |  |  |  |  |  |  |  |  |  |
| <5000 | 261(14.7) | 472(26.6) | 678(38.2) | 363(20.5) | 11.020 | 0.088 | 390(22.0) | 312(17.6) | 555(31.3) | 517(29.1) | 22.684 | **0.001** |
| 5000-8000 | 146(14.0) | 275(26.4) | 439(42.2) | 180(17.3) |  |  | 227(21.8) | 248(23.8) | 308(29.6) | 257(24.7) |  |  |
| >8000 | 21(20.2) | 27(26.0) | 43(41.3) | 13(12.5) |  |  | 28(26.9) | 25(24.0) | 31(29.8) | 20(19.2) |  |  |
| Years of work experience |  |  |  |  |  |  |  |  |  |  |  |  |
| <5 | 164(20.4) | 229(28.6) | 286(35.7) | 123(15.3) | 41.796 | **<0.001** | 218(27.2) | 178(22.2) | 212(26.4) | 194(24.2) | 42.505 | **<0.001** |
| 5-10 | 160(12.5) | 334(26.0) | 516(40.2) | 273(21.3) |  |  | 230(17.9) | 231(18.0) | 441(34.4) | 381(29.7) |  |  |
| >10 | 104(12.5) | 211(25.3) | 358(43.0) | 160(19.2) |  |  | 197(23.6) | 176(21.1) | 241(28.9) | 219(26.3) |  |  |
| Urban or rural hospitals |  |  |  |  |  |  |  |  |  |  |  |  |
| Urban | 274(15.5) | 466(26.4) | 700(39.6) | 326(18.5) | 3.068 | 0.381 | 415(23.5) | 382(21.6) | 525(29.7) | 444(25.1) | 17.773 | **<0.001** |
| Rural | 154(13.4) | 308(26.7) | 460(39.9) | 230(20.0) |  |  | 230(20.0) | 203(17.6) | 369(32.0) | 350(30.4) |  |  |
| Area |  |  |  |  |  |  |  |  |  |  |  |  |
| Eastern | 161(16.8) | 288(30.1) | 354(37.0) | 153(16.0) | 24.895 | **<0.001** | 222(23.2) | 189(19.8) | 295(30.9) | 250(26.2) | 22.674 | **0.001** |
| Central | 121(12.7) | 226(23.7) | 411(43.1) | 196(20.5) |  |  | 186(19.5) | 171(17.9) | 291(30.5) | 306(32.1) |  |  |
| Western | 146(14.5) | 260(25.8) | 395(39.2) | 207(20.5) |  |  | 237(23.5) | 225(22.3) | 308(30.6) | 238(23.6) |  |  |
| Total | 428(14.7) | 774(26.5) | 1160(39.8) | 556(19.1) |  |  | 645(22.1) | 585(20.0) | 894(30.6) | 794(27.2) |  |  |

**Table S3. Fatigue symptoms reported by respondents by work burdens**

| **Work burden** | **Sample size**, n (%) | **Fatigue symptoms** | | | | χ^2^ | P |
| --- | --- | --- | --- | --- | --- | --- | --- |
|  |  | Grade Ⅰ | Grade Ⅱ | Grade Ⅲ | Grade Ⅳ |  |  |
| A | 645(22.1) | 285(44.2) | 230(35.7) | 125(19.4) | 5(0.8) | 1333.197 | **<0.001** |
| B | 585(20.0) | 90(15.4) | 227(38.8) | 242(41.4) | 26(4.4) |  |  |
| C | 894(30.6) | 38(4.3) | 260(29.1) | 452(50.6) | 144(16.1) |  |  |
| D | 794(27.2) | 15(1.9) | 57(7.2) | 341(42.9) | 381(48.0) |  |  |
| Overwork in 1 month |  |  |  |  |  |  |  |
| Less or appropriate | 2063(70.7) | 399(19.3) | 639(31.0) | 787(38.1) | 238(11.5) | 403.483 | **<0.001** |
| Much | 674(23.1) | 26(3.9) | 118(17.5) | 306(45.4) | 224(33.2) |  |  |
| Very much | 181(6.2) | 3(1.7) | 17(9.4) | 67(37.0) | 94(51.9) |  |  |
| Irregular work (Such as sudden work, etc.) |  |  |  |  |  |  |  |
| Less | 2042(70.0) | 404(19.8) | 610(29.9) | 796(39.0) | 232(11.4) | 362.437 | **<0.001** |
| Much | 876(30.0) | 24(2.7) | 164(18.7) | 364(41.6) | 324(37.0) |  |  |
| Burden on business travel |  |  |  |  |  |  |  |
| No or less | 2741(93.9) | 423(15.4) | 756(27.6) | 1077(39.3) | 485(17.7) | 83.293 | **<0.001** |
| Much | 177(6.1) | 5(2.8) | 18(10.2) | 83(46.9) | 71(40.1) |  |  |
| Burden on working late at night |  |  |  |  |  |  |  |
| Less or appropriate | 1574(53.9) | 367(23.3) | 504(32.0) | 567(36.0) | 136(8.6) | 516.939 | **<0.001** |
| Much | 925(31.7) | 52(5.6) | 226(24.4) | 421(45.5) | 226(24.4) |  |  |
| Very much | 419(14.4) | 9(2.1) | 44(10.5) | 172(41.1) | 194(46.3) |  |  |
| Rest time or facilities |  |  |  |  |  |  |  |
| Satisfied | 1495(51.2) | 378(25.3) | 538(36.0) | 483(32.3) | 96(6.4) | 638.558 | **<0.001** |
| Dissatisfied | 1423(48.8) | 50(3.5) | 236(16.6) | 677(47.6) | 460(32.3) |  |  |
| The mental burden of work |  |  |  |  |  |  |  |
| Little | 1053(36.1) | 346(32.9) | 394(37.4) | 287(27.3) | 26(2.5) | 1130.462 | **<0.001** |
| Much | 1436(49.2) | 73(5.1) | 364(25.3) | 728(50.7) | 271(18.9) |  |  |
| Very much | 429(14.7) | 9(2.1) | 16(3.7) | 145(33.8) | 259(60.4) |  |  |
| The physical burden of work |  |  |  |  |  |  |  |
| Little | 1119(38.3) | 355(31.7) | 445(39.8) | 290(25.9) | 29(2.6) | 1170.856 | **<0.001** |
| Much | 1367(46.8) | 65(4.8) | 313(22.9) | 719(52.6) | 270(19.8) |  |  |
| Very much | 432(14.8) | 8(1.9) | 16(3.7) | 151(35.0) | 257(59.5) |  |  |
